# Successful Embolization of Posterior Inferior Pancreaticoduodenal Artery Pseudoaneurysm on the Grounds of Chronic Pancreatitis—Case Report and Literature Review

**DOI:** 10.3390/medicina56110617

**Published:** 2020-11-16

**Authors:** Milica Mitrovic, Vladimir Dugalic, Jelena Kovac, Boris Tadic, Stefan Milosevic, Borivoje Lukic, Nebojsa Lekic, Vladimir Cvetic

**Affiliations:** 1 Center for Radiology and Magnetic Resonance Imaging, Clinical Centre of Serbia, Pasterova No. 2, 11000 Belgrade, Serbia; dr_milica@yahoo.com (M.M.); jelenadjokickovac@gmail.com (J.K.); milosevic.stefan92@gmail.com (S.M.); radnicki@gmail.com (B.L.); radiovladacvetic@gmail.com (V.C.); 2 Department for HPB Surgery, Clinic for Digestive Surgery, Clinical Centre of Serbia, Koste Todorovica Street, No. 6, 11000 Belgrade, Serbia; vanjadug@yahoo.com (V.D.); nesalekic67@gmail.com (N.L.); 3 Department for Surgery, Faculty of Medicine, University of Belgrade, Dr Subotica No. 8, 11000 Belgrade, Serbia; 4 Department for Radiology, Faculty of Medicine, University of Belgrade, Dr Subotica No. 8, 11000 Belgrade, Serbia

**Keywords:** pancreatic pseudoaneurysm, chronic pancreatitis, pancreas, sandwich technique, angiography, minimally invasive treatment

## Abstract

Pancreatic pseudoaneurysm is a rare but life-threatening clinical entity. In this paper, we present a case of a 74-year-old man, who was admitted to our clinic with a diagnosis of an acute on chronic pancreatitis complicated by walled-off-pancreatic-necrosis, with subsequent development of peripancreatic pseudoaneurysm. After initial conservative management, the patient recovered and was discharged from the hospital. However, he soon returned feeling anxious due to a pulsatile abdominal mass. Abdominal Color–Doppler examination, CT scan, and angiography revealed large pancreatic necrotic collection in the total size of 9 cm, with centrally enhancing area of 3.5 cm that corresponded to pseudoaneurysm originating from the posterior pancreaticoduodenal vascular arcade. Considering the size, location of the pseudoaneurysm, feeding vessel, and poor general patients condition, we opted for minimally invasive treatment. Pseudoaneurysm was successfully managed by endovascular coil embolization, i.e., “sandwich technique”.

## 1. Introduction

Chronic pancreatitis (CP) is characterized by chronic, progressive inflammation of pancreatic parenchyma and fibrosis, irreversible destruction of the pancreas resulting in the loss of exocrine and endocrine function. A pseudoaneurysm, also known as a false aneurysm, is rare complication associated with chronic pancreatitis caused by erosion of the major pancreatic or peripancreatic vessels [1]. Lipolytic and proteolytic enzymes, that spread from the damaged pancreas during the disease, lead to elastolytic erosions of the wall of contiguous peripancreatic vessels, causing pseudoaneurysm formation. Such an enzyme eroded wall, combined with the pressure of an established pancreatic pseudocyst or walled-off-pancreatic-necrosis (WOPN), can convert into the pseudoaneurysm. The main clinical significance lies in its potential to rupture.

Although the incidence is estimated to be low (4–8%), bleeding from ruptured pseudoaneurysm can be potentially lethal with a mortality rate of up to 40% [2,3]. Timely diagnosis and early treatment are essential for the prevention of such a serious hemorrhagic complication. In this paper, we presented a patient with a rare pseudoaneurysm of a posterior inferior pancreaticoduodenal artery formed on the ground of chronic pancreatitis that was successfully treated with minimally invasive endovascular treatment, e.g., sandwich technique. Written informed consent was obtained from the patient.

## 2. Case Report

A 74-year-old man was admitted to our clinic with complaints of abdominal pain radiating to back, nausea, and vomiting of 7 days’ duration. The pain had a slow but progressive onset and started a few hours after alcohol consumption. His past medical history included chronic alcoholic pancreatitis, alcohol abuse, tobacco dependence, and hypertension. Five years earlier, due to occasional intense abdominal pain, the patient underwent a detailed gastroenterological examination and was diagnosed with alcohol related chronic pancreatitis. With enzyme therapy and lifestyle changes that included abstinence from alcohol and tobacco, and dietary recommendations, his condition was well and pain-free. The patient was also examined and followed by a psychiatrist with the aim of abstaining from alcohol. With benzodiazepine therapy, which focused primarily on anxiety and insomnia, a satisfactory therapeutic response has been achieved. For the past two years, the patient avoided psychiatric check-ups, continued to use alcohol, and stopped the therapy prescribed by the gastroenterologist.

At the time of admission, abdominal examination revealed diffuse tenderness, especially in the epigastric area. He was afebrile with normal vitals, blood pressure of 130/90 mmHg, and cachectic (BMI 17.4). Laboratory examinations, including complete blood count, revealed leukocytosis (white blood cells–14.6 × 109/L), elevated C-reactive protein and fibrinogen levels 96.2 mg/L and 4.5 g/L, respectively. Biochemistry test results were indicative of acute on chronic pancreatitis, with high amylase (2400 U/L) and lipase (2617 U/L) levels.

**Figure 1:**
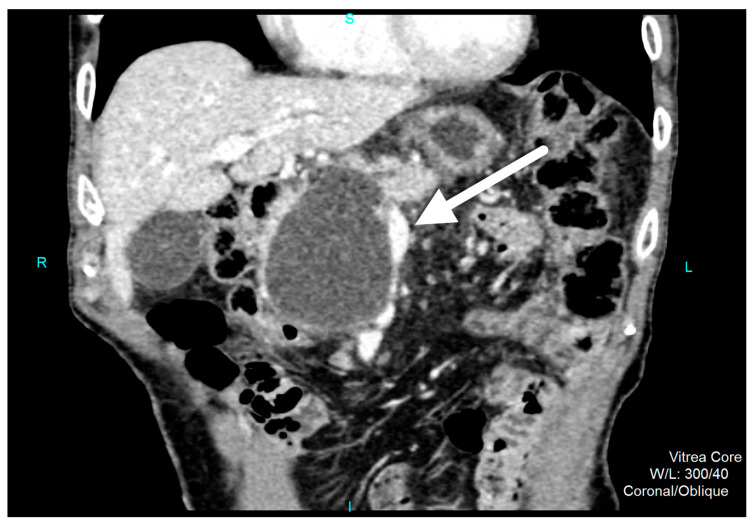
Coronal section contrast-enhanced abdominal computed tomography (CT) shows a well-defined round, encapsulated, necrotic collection that corresponds to walled-off-pancreatic-necrosis (WOPN), with a maximum diameter of 90 mm in the region of the pancreatic head and extrapancreatic extension into the duodeno-pancreatic space.

Since the initial abdominal ultrasound (US) was inconclusive due to patient meteorism, an abdominal contrast-enhanced computed tomography scan (CT) was done. It revealed encapsulated, round necrotic mass with a maximum diameter of 90 mm in the region of the pancreatic head with extrapancreatic extension into the duodeno-pancreatic space. An acute inflammation on the ground of the exacerbation of chronic pancreatitis, had a CT presentation of necrotic pancreatitis (Figure 1). The patient was treated conservatively with antibiotic prophylaxis (Imipenem 0.5 g, 8 hourly), high volume crystalloid infusion with Ringer’s lactate, analgesics (Tramadol 100–200 mg daily), and proton pump inhibitor (Pantoprasole 40 mg). On an abdominal ultrasound examination prior to patient discharge from the hospital, no worsening of inflammation, or new liquid collections, or the presence of free fluid was observed. The WOPN that was monitored seemed organized and uncomplicated.

Therefore, with normal values of inflammatory markers, the patient’s condition was considered stable and he was dismissed from the hospital on the 10th day upon admission.

**Figure 2:**
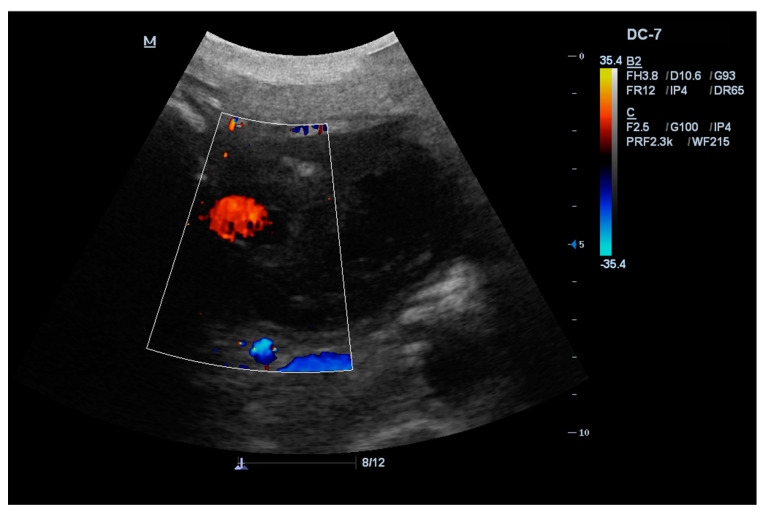
Abdominal ultrasound with color Doppler shows a large hypoechoic cystic lesion in the pancreatic head with internal turbulent blood flow.

**Figure 3:**
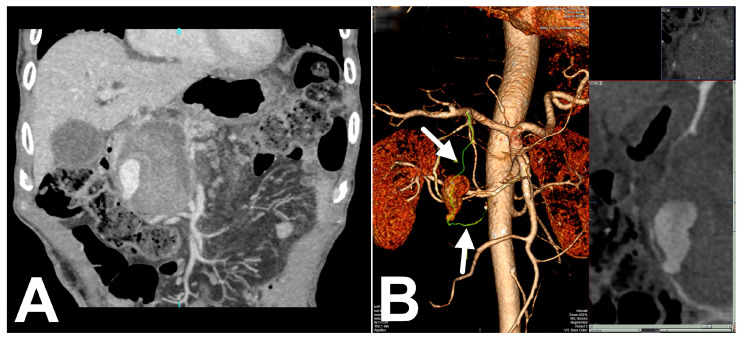
(**A**) Coronal section contrast-enhanced abdominal computed tomography (CT) demonstrates central enhancing component in previously verified walled-off pancreatic necrosis in the head of the pancreas. (**B**) 3D Volume Rendering CT shows central enhancing component surrounded with necrotic tissue and nearly formed hematoma that indicates pseudoaneurysm originating from the posterior inferior pancreaticoduodenal artery.

The patient arrived one week before the scheduled appointment, one month after hospitalization, feeling anxious due to a pulsatile mass in his abdomen. Physical examination found a soft, non-tender abdomen with a large palpable lump. Abdominal ultrasound showed a large hypoechoic cystic lesion in the pancreatic head. Color–Doppler examination revealed a turbulent blood flow in the cyst (Figure 2). Follow-up contrast-enhanced CT scan and angiography demonstrated central enhancing component in previously verified walled-off necrosis in the head of the pancreas (Figure 3A).

Area of contrast extravasation surrounded with necrotic tissue and nearly formed hematoma, was compatible with pseudoaneurysm originating from the posterior pancreaticoduodenal vascular arcade (Figure 3b). Considering the size, location of the pseudoaneurysm, and patient’s condition we opted for digital subtraction angiography (DSA) performed together with endovascular coil embolization.

**Figure 4:**
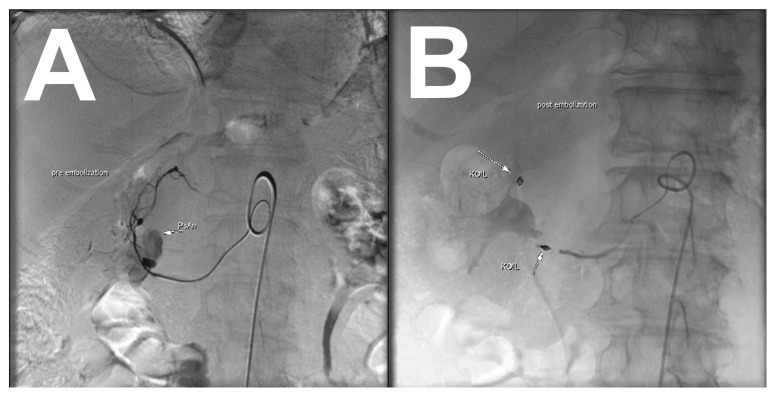
(**A**) Digital subtraction angiography (DSA) shows the pseudoaneurysm location at the pancreaticoduodenal arcade close to the superior mesenteric artery. (**B**) The sandwich technique was utilized, and both inflow and outflow of the pseudoaneurysm were embolized with coils (Concerto, Covidien, Irvine, CA, USA).

DSA confirmed the pseudoaneurysm location at the posterior inferior pancreaticoduodenal artery, close to the superior mesenteric artery (Figure 4A). The superior mesenteric artery had been subjected to selective catheterization with Sidewinder 2 (Optitorque, Terumo, Leuven, Belgium) which was followed by sub-selective catheterization of the posterior inferior pancreaticoduodenal artery, engaged with RebarTM14 microcatheter (ev3 Inc., Covidien, Irvine, CA, USA). The sandwich technique was utilized, and both inflow and outflow of the pseudoaneurysm were embolized with coils (Concerto, Covidien, Irvine, CA, USA). A control angiography demonstrated complete occlusion of the pseudoaneurysm (Figure 4B). The procedure was completed without complications and the patient was discharged from the hospital two days later.

**Figure 5:**
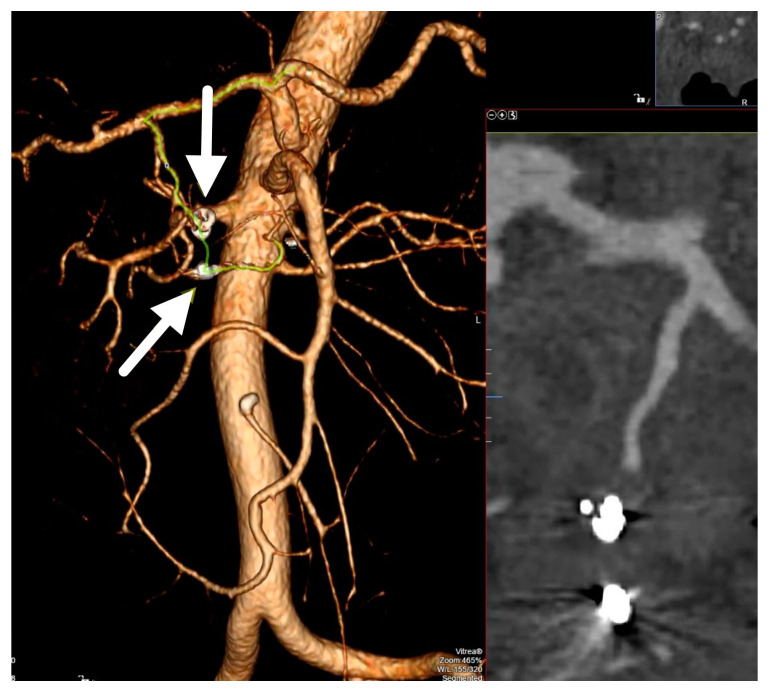
3D volume rendering CT image shows the complete exclusion of the pseudoaneurysm with coils.

Two months after the embolization, patient was reviewed. He was clinically stable. 3D volume rendering CT image showed complete exclusion of the pseudoaneurysm with coils (Figure 5).

## 3. Discussion

Chronic pancreatitis is a complex, multi etiological disease with various clinical presentations. The etiology of CP varies considerably due to differences in environmental exposure (alcohol and smoking) and genetic factors [4]. Most patients are treated with a diagnosis of alcoholic or idiopathic CP while the other causes also warrant consideration such as autoimmune diseases, cystic fibrosis, hypertriglyceridemia, congenital anomalies, pancreatic resection, and genetic defects. Although the underlying pathogenesis is not well understood, alcohol abuse is seen as the most common cause of CP and is diagnosed in 42% up to 77% of patients [5]. Recent research showed that genetic variants in CLDN2 gene loci influence the risk for alcohol related CP [6]. Such a genetic variant along with heavy alcohol consumption makes the pancreas more sensitive to injury and favor pancreatic disease progression [6].

Occasional acutization of this inflammatory process frequently leads to formation of the pancreatic fluid collections, pseudocysts, or walled-off pancreatic necrosis, in 20–40% patients [7,8].

During the course of an acute on chronic pancreatitis complicated with pancreatic or perpancratic necrosis, the initially formed acute necrotic collection gradually becomes organized and walled off. According to the revised Atlanta classification system, “walled off necrosis“ is defined as a collection associated with necrotizing pancreatitis, that persists after 4 weeks and develops a wall [9].

Each patient with WOPN should be treated individually and followed by a multidisciplinary team involving general surgeons, gastroenterologist, and radiologist [10]. Symptomatic or infected WOPN is commonly managed with antibiotics and percutaneous or endoscopic drainage, minimally invasive necrosectomy, or open surgery in advanced cases [11]. Complications may develop any time during the disease process until complete resolution of necrotic collection [12].

Pseudoaneurysm formation is found to be more common (80%) in patients where alcohol abuse is an underlying etiological factor [13,14]. During the process of formation and enlargement, a WOPN engages adjacent vessels into its wall. Unlike veins which mainly thrombose, thick-walled arteries continue to maintain their patency as they are exposed to the enzyme activity [1]. Once the visceral arterial wall integrity is damaged by the protein-lyzing enzymes, the pseudoaneurysm is formed [1]. Unlike true aneurysms, a pseudoaneurysm wall does not contain arterial tissue but consists of fibrous tissue and contains a hematoma, which can enlarge or rupture. The risk of rupture is higher than that of a true aneurysm of comparable size due to poor support of the aneurysm wall. Literature review shows that transformation of the pseudocyst or WOPN into a pseudoaneurysm is a relatively uncommon but potentially life-threatening complication, with an incidence of less than 10% but an overall mortality rate of 50% [1,15,16]. Profuse bleeding with hemodynamic instability due to the rupture or leak ranges from 1.4% to 8.4%, bearing the severe prognosis and high mortality rate up to 90% [17,18].

The most commonly involved vessels in pseudoaneurysm forming are the juxtapancreatic arteries, at first the splenic artery followed by the gastroduodenal artery and the pancreaticoduodenal arteries [19]. Sporadic cases of pseudoaneurysms of the aorta, celiac, hepatic, superior mesenteric, and gastric arteries have also been reported [20,21].

Non-bleeding pseudoaneurysms formed on the ground of chronic pancreatitis are mainly asymptomatic, but mild abdominal discomfort, pain, or palpable pulsating mass may occur [1]. In a case of bleeding, pseudoaneurysm is commonly manifested as anemia, melena, bleeding in the pancreatic duct (hemosuccus pancreaticus), bile duct (hemobilia), adjacent hollow organs, or massive hemorrhagia in the peritoneal cavity with severe abdominal pain and shock, requiring emergency laparotomy [1]. As the principal causal factor for chronic pancreatitis is long time alcohol abuse, the higher prevalence rate of gastrointestinal bleeding from erosive gastritis, peptic ulcer, or varices in these patients, and on the contrary, the rare occurrence of pancreatic pseudoaneurysm, leads to the fact that it is rarely considered in the differential diagnosis.

Taking into account the patient history of chronic pancreatitis, abdominal pain as the main symptom, and common abuse of painkillers, particularly uncontrolled self-administration of nonsteroid anti-inflammatory drugs, sudden bleeding from pseudoaneurysm can be mistaken for upper gastrointestinal bleeding, especially in emergency settings where actions are time-sensitive. However, 2.5% of bleeding pseudoaneurysms can be clinically inconspicuous [16].

As peripancreatic fluid accumulation can be expected after an episode of acute on of chronic pancreatitis, in addition to regular follow-up, patients should be monitored more intensively in the next few weeks. Large, well-demarcated peripancreatic fluid collections, over 5 cm in size, are commonly associated with complications. Pseudoaneurysm forming is the rarest but possibly lethal one, so every delay in management may lead to catastrophe [22].

US, Doppler US, CT, and angiography are all diagnostic modalities that can be used for the diagnosis of the pancreatic pseudoaneurysm. Abdominal contrast-enhanced CT scan is usually sufficient for appropriate identification of pancreatic pseudoaneurysms but it should be angiographically confirmed because this procedure is extremely valuable in localization of bleeding pseudoaneurysm [17].

At our center, as in the majority of centers, we follow a policy of monthly check-up of patients with WOPN, in the form of clinical and laboratory assessments as well as abdominal US/MSCT since the size of WOPN shows a significantly decreasing trend. In our case, the pseudoaneurysm appeared as a complication immediately after the discharge of the patient from the hospital, which implies that clinicians must be very careful in monitoring these patients and that monthly follow up might be insufficient.

Surgical treatment of pancreatic pseudoaneurysm has been linked with a high mortality rate [2]. Therefore, it is reserved only for hemodynamically unstable patients, who are in a life-threatening condition, and where angiography would represent a waste of valuable time to save the patient’s life. Fibrosis of peripancreatic tissue, which can be seen in chronic pancreatitis, makes surgical treatment even harder and increases the possibility of lethal outcome. Pancreatic resections, especially in emergency settings should be avoided, instead of it, ligation of the vessel that feeds the pseudoaneurysm is the most appropriate treatment option [2]. When all other options are exhausted, pancreatic resection along with the pseudoaneurysm is the only choice.

Modern interventional radiology provides extraordinary diagnostic and therapeutic possibilities for precise identification and localization of a blood vessel that is engaged in the formation of the pancreatic pseudoaneurysm. Furthermore, the exclusion of that blood vessel from circulation by stenting or embolization methods is possible at the same time. Although there are no randomized prospective studies, numerous reports have shown excellent results of endovascular treatment in this group of high-risk patients. To date, angioembolization has been considered the most appropriate treatment option for treatment of visceral pseudoaneurysms associated with pancreatitis and the power tool for achieving hemostasis [14,18]. When endovascular embolization is not possible due to anatomical reasons, thrombin injection, as a cheaper but more invasive procedure, may be the method of choice. Thrombin injection is suitable for pseudoaneurysms with a narrow neck, while angioembolization is recommended for those with wide neck [14,23].

## 4. Conclusions

Pseudoaneurysm, as a rare complication of chronic pancreatitis, is characterized by a high mortality rate and therefore requires prompt diagnosis and treatment. It may occur at any time due to acute infection or due to the formation of necrotic collections and for this reason, care should be taken in monitoring and treating these patients. Angioembolization, endovascular stenting, and percutaneous thrombin injection represent effective and minimally invasive therapeutic options and the gold standard in the treatment of these patients.
